# Genome-guided identification of novel head-to-tail cyclized antimicrobial peptides, exemplified by the discovery of pumilarin

**DOI:** 10.1099/mgen.0.000134

**Published:** 2017-09-25

**Authors:** Auke J. van Heel, Manuel Montalban-Lopez, Quentin Oliveau, Oscar P. Kuipers

**Affiliations:** ^1^​ Molecular Genetics, University of Groningen, Groningen, Nijenborgh 7, NA 9747 AG, The Netherlands; ^2^​ Department of Microbiology, University of Granada, Granada, Spain

**Keywords:** genome-mining, bacteriocin, circular peptide, antimicrobial peptide, annotation

## Abstract

The need for novel antibiotics in an era where antimicrobial resistance is on the rise, and the number of new approved antimicrobial drugs reaching the market is declining, is evident. The underused potential of post-translationally modified peptides for clinical use makes this class of peptides interesting candidates. In this study, we made use of the vast amounts of available genomic data and screened all publicly available prokaryotic genomes (~3000) to identify 394 novel head-to-tail cyclized antimicrobial peptides. To verify these *in silico* results, we isolated and characterized a novel antimicrobial peptide from *Bacillus pumilus* that we named pumilarin. Pumilarin was demonstrated to have a circular structure and showed antimicrobial activity against several indicator strains, including pathogens.

## Abbreviations

CID, collision-induced dissociation; TFA, trifluoroacetic acid.

## Data Summary

Four supplementary tables and five supplementary figures are available in four supplementary files, three of which can be found online in Figshare at https://figshare.com/s/d318500f36414b4ccfe5 and the other one can be found in the online Supplementary Material. All supporting data, code and protocols have been provided within the article or through supplementary data files or public repositories.

## Impact Statement

Genome mining approaches are powerful in identifying novel natural products, reducing the search space for novel molecules and focusing screening efforts. In this study, we focused specifically on the intriguing group of head-to-tail cyclized antimicrobial peptides. These circular bacteriocins are post-translationally modified so that their N- and C-termini are fused. This cyclized structure can make proteins more resistant to degradation. The *in silico* mining efforts not only resulted in many newly identified peptides, but also yielded a new active antimicrobial we named pumilarin. Characterization of this compound also confirmed the predicted circular structure.

## Introduction

The vast class of post-translationally modified peptides demonstrates a wide variety of chemical structures present in them [1]. These peptides are highly interesting not only due to their diverse biological activities but also due to their different chemical structures that often result in improved activity and stability. An interesting group of ribosomally synthesized and post-translationally modified peptides is the one encompassing bacterial head-to-tail cyclized peptides, commonly referred to as circular bacteriocins [2–4]. These circular bacteriocins arise after the cleavage of a leader peptide and the formation of a peptide bond between the N- and C-termini. Little is known yet about the circularization mechanism of circular bacteriocins compared to other smaller circular peptides coming from ribosomal synthesis, such as cyclotides, cyanobactins or pilins [5]. Nevertheless, the presence of a circular backbone clearly shows an increased stability and antimicrobial activity compared to linear counterparts [6–8]. A renewed interest in bacteriocins and post-translationally modified peptides has emerged due to their potential in therapeutics [9–12] and the utility that some of the enzymes can have in the stabilization of biologically active peptides [13, 14]. Although diverse chemical and enzymatic processes have been studied for the production of circular proteins, this is still challenging, and natural discovery and production remains the main source of novel circular peptides [15, 16].

Circular bacteriocins characterized so far display a saposin-fold, which is similar to the structure of leaderless bacteriocins [4, 17, 18]. Two main subclasses are suggested based on structural and biochemical characteristics. Subclass I encompasses larger strongly cationic peptides with four or five helices, whereas subclass II includes acidic circular bacteriocins [19]. The antimicrobial activity of circular bacteriocins is based on pore formation, although the mechanisms involved are different [4]. Carnocyclin A is a monomeric peptide that creates pores in a voltage-dependent manner [20, 21], whereas AS-48 is present in solution as a dimeric bacteriocin that does not require voltage to create toroidal pores [22, 23]. Recently, a maltose transporter has been suggested to increase the sensitivity to garvicin ML [24].

**Fig. 1.**
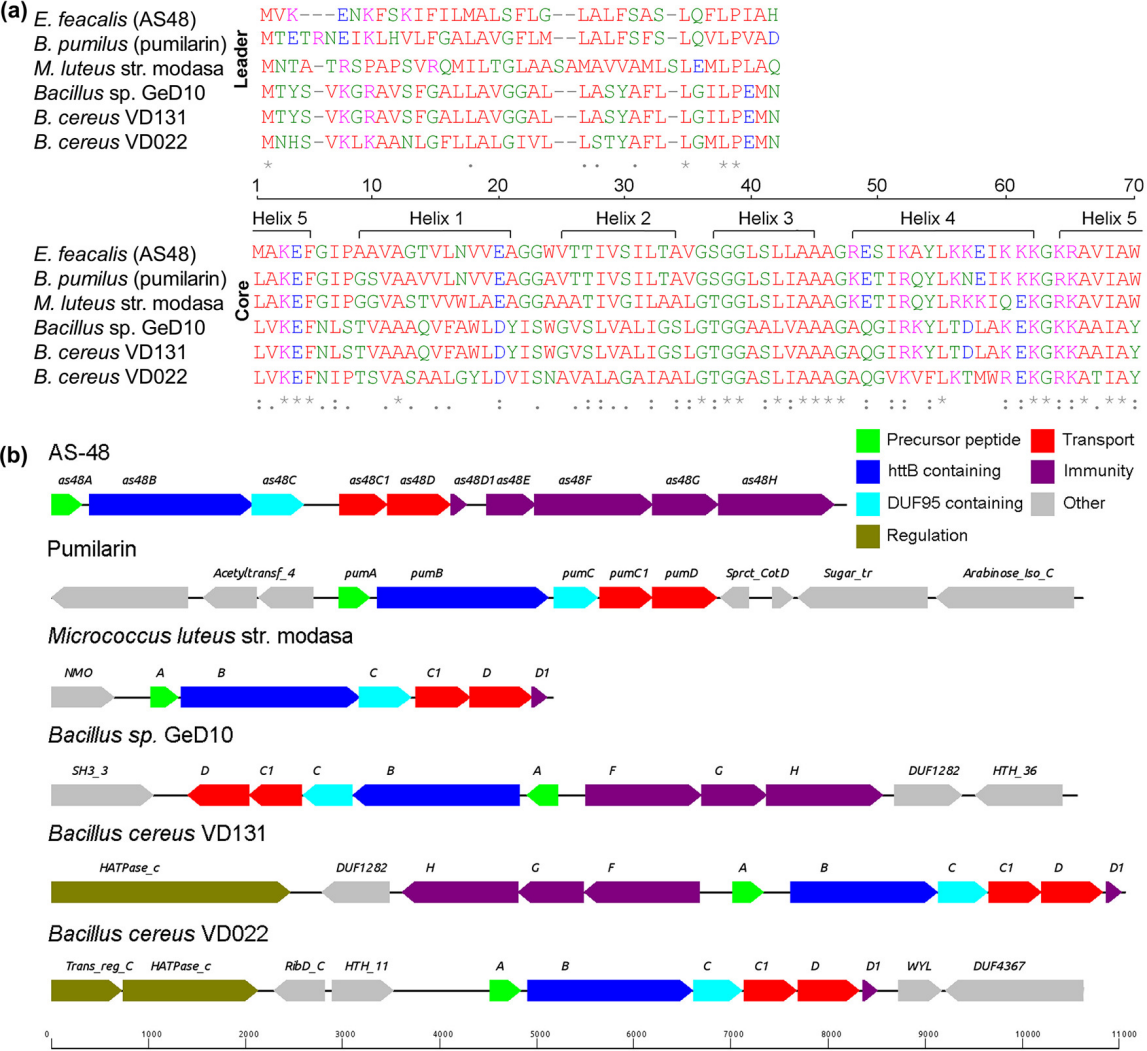
(a) Alignment of peptides identified with bagel3 that show similarity to enterocin AS-48 (top, leader peptides; bottom, core peptides). Pumilarin was identified *in silico* in *B. pumilus* B4107, *B. pumilus* BA06 BA_1113 (NZ_AMDH01000012.1, WP_017358050.1), *B. pumilus* S-1 scaffold28 (NZ_JH137674.1, WP_017358050.1), *Bacillus* sp. M 2–6 strain KACC 16563 241_9 (leader T2A) (NZ_AJWW01000009.1, WP_008342549.1). An asterisk (*) indicates a single, fully conserved residue. A colon (:) indicates conservation within groups of residues with strongly similar properties. A period (.) indicates conservation within groups of residues with weakly similar properties. (b) Gene clusters responsible for the production of (putative) head-to-tail cyclized peptides. Image created using Genome2d (http://genome2d.molgenrug.nl). Genes are named according to the AS-48 nomenclature, other names that are indicated are the pfam names of the pfam domains with the best hits.

The prototype of circular bacteriocins is the enterocin AS-48, produced by *Enterococcus faecalis* subsp. *liquefaciens* S-48 [25, 26]. AS-48 has a broad antimicrobial spectrum targeting mostly Gram-positive bacteria such as *Listeria*, *Bacillus*, *Enterococcus* and *Staphylococcus*, but also some Gram-negative micro-organisms such as *Escherichia coli* or *Salmonella* [25]. The genes encoding proteins that are responsible for the production of and resistance to AS-48 are located on the conjugative plasmid pMB2 (see Fig. 1b) [27]. AS-48 and natural variants of AS-48 have been found in other enterococcal species receiving diverse names: bacteriocin 21 [28], enterocin 4 [29], EFS2 [30], UGRA10 [31], RM6 [32] and AS-48RJ [33], which is so far the only variant that is not plasmid based. The presence of AS-48 in enterococcal strains of such a diverse origin points at a wide distribution of AS-48 variants in nature.

The production of AS-48 requires the coordinated expression of 10 genes arranged in three operons, although some ambiguities exist in the literature regarding the length of the ORFs and the promoters regulating the production [4, 27, 28, 34, 35]. In addition to the structural gene *as-48A*, there are two transporters, *as-48C_1_D* and *as-48EFGH*, involved in production and immunity, respectively, an immunity protein, *as-48D_1_*, and two additional ORFs, *as-48B* and *as-48C* [2, 26]. Notably, homologues of As-48C containing a so called SpoIIM (PF01944, previously known as DUF95) motif are found in other head-to-tail cyclized bacteriocin clusters [19], this domain is related to both immunity and circularization [36].

In recent years, diverse bioinformatics tools have been developed to mine genomic sequences for clusters encoding ribosomal and non-ribosomal natural products [37, 38], and even to link genotype to phenotype using high-resolution techniques such as MS in a high-throughput manner [39]. These tools, in combination with cheap and fast DNA sequencing, can help focus research efforts on strains that actually encode the capability of producing new antimicrobial compounds [40]. Genome mining has proved to be efficient for the identification of novel molecules, such as the lantibiotics lichenicidin [41] and prochlorosins [42]. Here we report the results from *in silico* mining for novel head-to-tail cyclized peptides using bagel3 on all publicly available genomes (hosted on the National Center for Biotechnology Information site; http://ftp.ncbi.nlm.nih.gov). A total of 394 potential clusters were identified that encoded 59 unique peptides. To validate our results, we focused our experimental efforts on a new cluster found in *Bacillus pumilus* B4107 [43]. This resulted in the identification, production, purification and characterization of a novel head-to-tail cyclized antimicrobial peptide we named pumilarin, which is, to the best of our knowledge, the first of its kind in *B. pumilus*.

## Methods

### Genome mining for head-to-tail cyclized peptides

bagel3 [38] was run on all public prokaryotic genomes found on the National Center for Biotechnology Information ftp server (https://ftp.ncbi.nlm.nih.gov/genomes/refseq/bacteria/) (October 2013). This involved roughly 3000 genomes. The results were filtered to only contain clusters identified based on the SpoIIM (PF01944) domain (previously known as DUF95). The PF01944 domain was chosen because it is the least common but conserved domain that is present in the biosynthesis gene clusters of head-to-tail circularized bacteriocins. Next, only clusters in which a potential prepeptide could be identified were selected. The results were organized according to homology to known head-to-tail cyclized peptides.

### Strains and media

The strains used in this study are listed in Table S1 (available in the online Supplementary Material). *Bacillus* strains, *B. pumilus* B4107, *Micrococcus flavus* and *Listeria monocytogenes* were grown in LB (Oxoid) at 30 °C with vigorous shaking (200 r.p.m.). The other bacteria listed were grown at 37 °C in M17 medium (Oxoid) supplemented with 0.5 % glucose (GM17). *B. pumilus* B4107 was grown on complex medium for bacteriocin production and isolation [44].

### Bacteriocin purification

The purification of pumilarin was performed using cationic exchange and reversed-phase chromatography. The protocol was slightly modified from that of Abriouel *et al.* [45]. Briefly, a 2 l flask containing 0.5 l complex medium was inoculated 1 : 20 with an overnight culture of *B. pumilus* B4107 and was grown for 8 h (30 °C, shaking at 200 r.p.m.) after which rehydrated CM Sephadex C-25 resin (GE Healthcare) was added to the culture in a 1 : 40 ratio (v/v). The mixture was incubated for 1 h with shaking to allow binding of the cationic compounds to the resin and subsequently left standing for 1 h (so that the resin settled). The top of the mixture was decanted into a waste container and the rest (containing the resin) was packed into a glass column. The column was washed with three volumes of washing buffer (20 mM phosphate buffer pH 6.5). Next, pumilarin was eluted from the column with five volumes of elution buffer (1.5 M NaCl, 20 mM phosphate buffer pH 6.5). Pumilarin was further purified and desalted using reversed-phase chromatography in an open C18 (Spherical C18 Supelco) column. The resin was equilibrated in 0.1 % trifluoroacetic acid (TFA) prior to the application of the samples. The elution was performed with three elution steps using three volumes of 33, 66 and 100 % organic solvent (isopropanol : acetonitrile 2: 1 0.1 % TFA). All the steps were monitored using activity tests. Pumilarin was purified to homogeneity by HPLC in an Agilent 1260 series chromatographer using an Aeris peptide 3.6 u XB-C18 250×4.6 mm column and 0.1 % TFA dissolved in isopropanol : acetonitrile 2 : 1 as the mobile phase. The peptide was injected to the column equilibrated in 5 % organic solvent. The concentration of organic solvent increased in 5 min to 60 % and the peptide eluted in a gradient going up to 70 % organic solvent in 15 min.

### MS and fingerprinting

Matrix-assisted laser desorption/ionization-time of flight (MALDI-TOF) MS analysis was performed as described elsewhere [46] using a Voyager DE Pro mass spectrometer. Determination of mass and circularity was performed as described elsewhere [32]. Briefly, 5 µl of a HPLC fraction containing the intact peptide was directly infused using static nanospray with a 2 µm tip (Picotip; New Objective). Survey scans were recorded using a Thermo Scientific LTQ Orbitrap XL at a FT resolution of 60 000 at *m*/*z* 400 using a 1.3 kV spray voltage for 2 min. The major charged signal of the 7 KDa peptide at *m*/*z* 1418 (5+) was selected for collision-induced dissociation (CID) at 35 % in the iontrap and analysed in the Orbitrap at a resolution of 6000 for 5 min. Internal fragment masses were calculated using MS-product from Prospector (http://prospector.ucsf.edu/prospector/cgi-bin/msform.cgi?form=msproduct). Fragments were considered as detected when the mass matched the theoretical mass by at least two decimal precision.

### Proteolysis assays

HPLC purified and lyophilized pumilarin was resuspended in 50 mM Tris, 5 mM CaCl_2,_ pH 7.8 and incubated at 37 °C for 4 h in the presence of sequencing grade trypsin, proteinase K or ɑ-chymotrypsin. Proteolysis using pepsin was performed in 0.04 M HCl at 37 °C for 4 h and the reaction with protease XIV or endoprotease Glu-C was conducted in 50 mM ammonium bicarbonate pH 8.0 at 37 °C for 4 h. All the enzymes were provided by Sigma-Aldrich. They were dissolved in the reaction buffer at 1 mg ml^−1^ and added at a ratio enzyme:substrate 1 : 50. BSA (1 mg ml^−1^) was used as a control. The cleavage was monitored by analytical HPLC as described elsewhere [8].

### Activity tests

In order to test the production of pumilarin in solid media, 5 µl overnight culture of *B. pumilus* B4107 was spotted on minimal expression medium [47], GM17, LB, brain heart infusion broth or complex medium [44]. It was incubated overnight at 30 °C. The culture was overlaid with 6 ml GM17 0.75 % agar buffered with 0.1 M phosphate buffer (pH 7.0) and inoculated with 200 µl overnight culture. The overlay was grown until the inhibition halos were visible.

The spot-on-lawn technique was used to monitor the purification steps and to compare the inhibition of pumilarin and the enterocin AS-48, used as a reference. In this case, the appropriate solid medium was melted and inoculated at 1 % (v/v) with an overnight culture of the sensitive strain. The plates contained an equal volume (13 ml) of melted agar with inoculum. A total of 5 µl of a purified bacteriocin dissolved in 20 mM phosphate buffer pH 6.5 was spotted on top of the agar. The plate was incubated until the inhibition halos were visible. The experiment was conducted in duplicate. The concentration of the samples was determined by measuring the absorbance in the UV range using the molar extinction coefficient calculated with http://web.expasy.org/protparam/.

To determine the MIC for pumilarin growth assays were performed using a 96 well plate reader (Tecan infinite F200). To every well, 135 µl appropriate medium with 1 % (v/v) inoculum and 15 µl two-fold bacteriocin dilution, quantified as indicated above, was added (total volume 150 µl). The OD_600_ was monitored for 16 h under appropriate growth conditions. The indicator strain was grown overnight, diluted 1 : 50 in the morning and grown until it reached mid-exponential growth prior to addition.

### Secondary structure prediction

The secondary structure prediction of the head-to-tail cyclized bacteriocins was performed on the psipred server [48, 49].

## Results

### Identification of 394 gene clusters encoding head-to-tail cyclized bacteriocins

In an effort to gain insight into the prevalence of head-to-tail cyclized antimicrobial peptides and to find new ones, we screened all publicly available genomes using bagel3. We identified 394 genetic clusters potentially encoding a head-to-tail cyclized peptide [see Table S2 (https://figshare.com/s/d318500f36414b4ccfe5)]. These 394 clusters encoded 59 unique sequences that had at least one amino acid difference in the leader or core peptide. All 394 clusters contained at least a potential prepeptide and a protein encoding a PF01944 domain, which is also found in the As-48C predicted protein and other sequenced head-to-tail cyclized bacteriocin gene clusters [36, 50]. A total of 43 of the 394 gene clusters contained a prepeptide with homology to a known circular bacteriocin. One cluster identified in diverse strains of *Staphylococcus aureus* was conserved (100 %identity at the amino acid level of the prepeptides) in 230 of the 473 sequenced strains at the time of the bagel3 run. Another conserved cluster was found in 53 of the 275 sequences of *Streptococcus pneumoniae.* Additionally, we found four other clusters in the genus *Streptococcus*. In *Oenococcus oeni*, we found at least one cluster in 12 out of the 15 sequenced strains. Of these 12 strains, 8 contained two or more head-to-tail cyclized bacteriocin clusters. In the genus *Lactobacillus,* we identified five clusters mostly (four out of five) showing weak homology to known clusters. In the genus *Geobacillus,* we identified five clusters all showing weak homology to the circularin A cluster. In diverse *Bacillus* species, we found 53 clusters, of which 27 were unique and 15 of which the prepeptides showed (weak) homology to known circular bacteriocins. Furthermore, we identified 12 clusters in other bacteria. We generated alignments (using ClustalW), aligning the newly identified circular peptides to known circular peptides. This resulted in alignments for four subclasses: enterocin AS-48-like (see Fig. 1), butyrivibriocin-like (see Fig. S1), circularin A-like (see Fig. S2) and uberolysin-like (see Fig. S3). A figure of the taxon distribution can be found in the supplementary information (see Fig. S4) All full protein sequences and accession numbers can be found in Table S2.

The AS-48-like class alignment (Fig. 1a, top) of putative leaders only shows strong homologies at their C-terminal part. At the −8 to −4 position, a L**LP motif is clearly visible. In spite of the low sequence homology, the secondary structure prediction shows a large helix in the leader peptide that is disrupted by the L**LP motif in all cases and a small helix formed by the end of the leader peptide and the first residues in the core peptide (Fig. S4). The core peptides (Fig. 1a, bottom) contain several strongly conserved regions. At the start of the core peptides a (L/M)(A/V)KEF(G/N)(I/L) motif can be observed. At position 20, a negatively charged amino acid is present in all cases (E/D). Furthermore, all residues involved in helix 4 (residues 36–47; see Fig. 1a) are very conserved in all the variants. Another conserved region covers the end of helix 4 and the beginning of helix 5 (residues 60 to 70; see Fig. 1a). Thus, it is important to note that both the N- and the C-termini that are linked during biosynthesis are conserved.

The gene clusters all have a different composition when compared to the gene cluster of AS-48. Although all clusters do have the main part (homologues to As-48ABCC_1_D), responsible for production (a*s-48A*, *as-48B* and *as-48C*) and transport (*as-48C_1_D*), in common, the immunity associated genes are sometimes lacking or present in an opposite direction (*as-48D_1_* and *as-48EFGH*). Notably, the *as-48E* gene was not identified in any of the other clusters. Another interesting observation is the fact that no specific immunity related genes were identified in the pumilarin cluster. The typical small cationic hydrophobic protein (D_1_ in AS-48) could not be identified in the gene clusters of pumilarin and *Bacillus* sp. GeD10. In the cluster identified in *Micrococcus luteus*, no homologues of the immunity-associated transporter As-48EFGH were identified, but this might be due to the fact that the DNA contig ends after the As-48D_1_ homologue. Nevertheless, in an *in silico* study on streptococcal species, a prevalent putative gene cluster encoding circular bacteriocins was found that does not contain an immunity-related transporter either.

### *B. pumilus* B4107 produces a novel circular bacteriocin: pumilarin

**Fig. 2.**
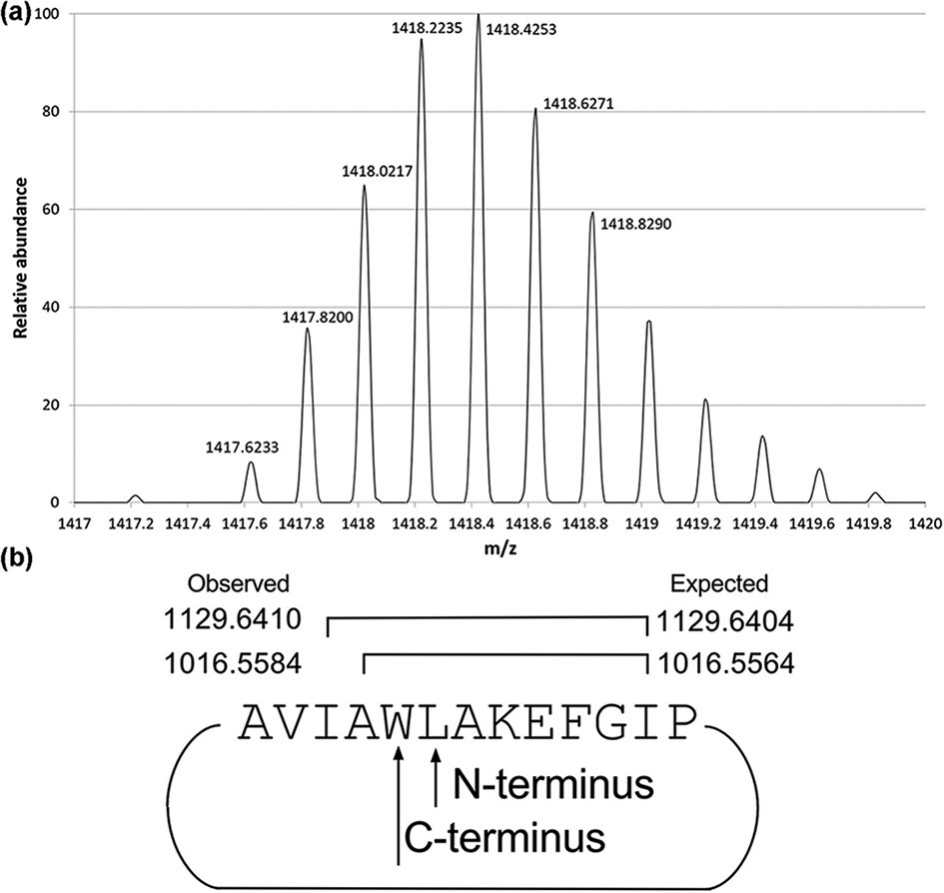
(a) The exact mass determination of pumilarin shown here is the (most prevalent) five times charged (since the difference between the isotope peaks in *m*/*z* is 0.2) variant. The theoretical mass of the protein is 7083.0694 (7105−18 the mass that is lost during cyclization), which is in line with the observed mass 7083.0768 [1417.6233×5 (five times charged)=7088.9−(5×1.00794)=7083.0768]. (b) Observed fragments connecting the N- and C-termini. The active purified protein pumilarin was further analysed by MS/MS. After the first MS step, pumilarin was selected and subsequently fragmented by CID and the mass of the resulting fragments was recorded in the second MS step. Depicted here are the observed fragments that contain the N- and C-termini of the antimicrobial protein in line with the circular nature of the compound (for full spectra see Tables S3 and S4; https://figshare.com/s/d318500f36414b4ccfe5).

To validate our *in silico* screening results, we focused our attention towards the AS-48-like clusters we identified. We choose this group because of strain availability and because we had experience working with AS-48. We attempted to purify and characterize the circular bacteriocin identified in *B. pumilus* B4107, which we called pumilarin. First, we attempted the production in different solid media, monitoring it by activity tests against *Micrococcus flavus*, *Listeria monocytogenes* and *Enterococcus faecalis* JH2-2. In all cases, after the incubation we could detect an inhibition zone around *B. pumilus* B4107 colonies (data not shown). These results were indicative that *B. pumilus* B4107 was producing an inhibitory agent. In order to determine whether that agent is the predicted head-to-tail cyclized peptide, we purified cationic hydrophobic proteins from 1 l supernatant of a *B. pumilus* culture according to predicted physico-chemical properties. The approximate yield of the overall purification process rendered roughly 100–200 µg HPLC-grade pumilarin (l supernatant)^−1^ HPLC-grade pumilarin. Large variation in the yield was observed in the different batches purified. The HPLC purified peptide that retained activity was analysed by a Thermo Fisher Orbitrap (Fig. 2a) showing a mono isotopic peak of ~1417.6233 Da, which corresponds to a five times [*m*/*z* difference between the different isotope peaks is 0.2 (1/0.2=5)] ionized peptide. The observed mono-isotopic mass of this peptide is, therefore, 7083.0768 Da [=(5×1417.6233)–5.0397], this 0.0074 Da less than the expected mono-isotopic mass of the whole peptide 7083.0694 [=7101.08 −18.010565 Da (cyclization)]. The loss of approximately 18 Da that was observed is consistent with a dehydration derived from the peptide bond formation between the N-terminal Leu and the C-terminal Trp. To unambiguously prove this assumed circularity, we performed CID to fragment the peptide. Among the observed fragments there were masses (1129.64, 1016.56 Da) perfectly corresponding to peptide fragments containing both the C-terminal Leu and the N-terminal Trp (AVIA**WL**AKEF/IA**WL**AKEFGI, IA**WL**AKEFG/A**WL**AKEFGI) (see Fig. 2b). In addition, trypsin and other proteases were tested to prove the peptidic nature of the inhibitory compound and its resistance against proteolysis (Fig. S5). Pumilarin showed high resistance to proteolysis, similarly to previous reports on AS-48. After 4 h digestion the non-specific proteases ɑ-chymotrypsin, pepsin, protease XIV and proteinase K were able to significantly degrade the protein, whereas the more specific proteases endoprotease Glu-C and trypsin did not have a pronounced effect.

### Pumilarin has a broad antimicrobial spectrum including Gram-negative targets

Purified pumilarin was tested against a panel of Gram-positive and Gram-negative bacteria including foodborne pathogens and clinically relevant multidrug resistant strains, such as *Staphylococcus aureus* and *Enterococcus faecalis*. The activity of pumilarin was compared in the test with that of the reference head-to-tail cyclized bacteriocin AS-48, and the data are summarized in Tables 1 and 2. In a more qualitative spot-on-lawn antimicrobial assays (see Table 1), pumilarin displayed an activity weaker than that of AS-48 against all the strains used, except against *E. coli* where pumilarin produced a clearance. Both bacteriocins were particularly active against the *Bacillus* strains analysed. The activity of pumilarin against meticillin-resistant *Staphylococcus aureus* (strains CAL and MW2) and *Enterococcus faecalis* was also reduced. Notably, the producer strain *B. pumilus* B4107 was sensitive to both AS-48 and pumilarin using relative high amounts of peptide.

**Table 1.** Comparison of the antimicrobial activity of pumilarin and AS-48 by spot-on-lawn technique The concentration of the samples was determined by measuring the absorbance under UV. *E. faecalis, Enterococcus faecalis; L. lactis*, *Lactococcus lactis*; *M. flavus*, *Micrococcus* *flavus*; *M. smegmatis*, *Mycobacterium smegmatis*; *S. aureus*, *Staphylococcus* *aureus*.

**Sensitive strain**	**Bacteriocin**	**230 µg ml^−1^**	**57.5 µg ml^−1^**	**14.4 µg ml^−1^**	**3.6 µg ml^−1^**
*B. cereus* ATCC14579	AS-48	+	+	−	−
	Pumilarin	+	+	−	−
*B. pumilus* B4107	AS-48	+	+	−	−
	Pumilarin	+	+/−	−	−
*B. pumilus* KMM62	AS-48	+	+	−	−
	Pumilarin	+	+	−	−
*B. subtilis* 168	AS-48	+	+	−	−
	Pumilarin	+	+/−	−	−
*E. faecalis* JH202	AS-48	+	+	−	−
	Pumilarin	−	−	−	−
*E. faecalis* V538	AS-48	+	+	−	−
	Pumilarin	−	−	−	−
*E. faecalis* VE14089	AS-48	+	+	−	−
	Pumilarin	−	−	−	−
*E. coli* DH5*α*	AS-48	−	−	−	−
	Pumilarin	+/−	−	−	−
*L. lactis* NZ9000	AS-48	+	+	−	−
	Pumilarin	+	−	−	−
*M. flavus B423*	AS-48	+	−	−	−
	Pumilarin	+	−	−	−
*M. smegmatis*	AS-48	−	−	−	−
	Pumilarin	−	−	−	−
*S. aureus* CAL	AS-48	+/−	−	−	−
	Pumilarin	−	−	−	−
*S. aureus* MW2	AS-48	−	−	−	−
	Pumilarin	+/−	−	−	−

**Table 2.** Comparison of the MIC of pumilarin and AS-48 determined by broth microdilution assay The concentration of the samples was determined by measuring the absorbance under UV. *M. flavus*, *Micrococcus* *flavus*; *S. pneumoniae*, *Streptococcus* *pneumoniae*.

Strain	MIC pumilarin (μg ml^−1^)	MIC AS-48 (μg ml^−1^)
*B. cereus* ATCC14579	12	>12
*B. pumilus* B4107	12	12
*E. coli* DH5α	12	>47
*M. flavus* B423	3	>12
*S. pneumoniae* D39	47	3

In antimicrobial activity assays performed in liquid (Table 2), pumilarin showed strong activity against *Micrococcus flavus* B423 (MIC 2.9 µg ml^−1^), and intermediate activity against *Bacillus cereus* ATTC14579, *B. pumilus* B4107 and *E. coli* DH5α (MIC 11.75 µg ml^−1^). Weak activity was observed against *Streptococcus pneumoniae* D39 (MIC 47 µg ml^−1^). Pumilarin outperformed AS-48 in three cases (*B. cereus* ATCC14579, *E. coli* DH5α and *Micrococcus flavus*). In one case, the determined MIC was identical (*B. pumilus* B4107). AS-48 showed a much lower MIC against *Streptococcus pneumoniae.*

## Discussion

The fact that almost 400 head-to-tail cyclized bacteriocin clusters were identified in the approximately 3000 screened strains highlights their widespread occurrence. However, only 11 different head-to-tail cyclized bacteriocins have been isolated from nature to date [3, 51]. The large number of identified clusters might be biased due to large number of *Staphylococcus aureus* and *Streptococcus pneumoniae* sequences that have been screened and the frequent occurrence of circular peptides in these strains. Roughly half of the unique prepeptides were identified in the genus *Bacillus*, indicating a high diversity of head-to-tail cyclized bacteriocins in this genus. Almost all clusters were identified in genomes within the phylum Firmicutes, except for five clusters found in propionibacteria and notably one cluster found in the Gram-negative lignin-degrading *Novosphingobium* sp. B-7. The identification of the gene cluster of pumilarin and other head-to-tail cyclized peptides by genome mining provides also valuable information regarding conserved residues in the peptide sequence that might play a role in the recognition by the yet unidentified maturation enzymes and in peptide circularization. The role of the different residues involved in the leader peptide cleavage and circularization was studied in enterocin AS-48 [52]. Most circular bacteriocins, including pumilarin (Fig. 1), carry an aromatic residue in the C-terminal position (either Trp or Tyr) and, therefore, it has been hypothesized that they play an important role during cyclization [5, 26, 52]. In fact, the mutant W70A of the enterocin AS-48 displayed impaired cyclization activity that allowed the purification of a linear form of AS-48. The first amino acid in the propeptide is also well conserved, being in most cases a Leu, except for AS-48, which has a Met. The leader peptide of diverse ribosomally and post-translationally modified peptides (RiPPs) has been shown to be a recognition/activation sequence for the modification machinery, a secretion signal and a way to keep the prepeptide inactive during the production [53, 54]. However, there is no clear homology between the leader peptides of the different circular bacteriocins, which range between 35 to 3 amino acids [26]. The leader peptides show more sequence variability. Only a conserved L**LP motif in the C-terminal part of the leader is conserved in the circular bacteriocins with an extended leader peptide such as AS-48 and pumilarin. The presence of a conserved proline at the −4 position could disrupt the helical conformation predicted for the leader peptide of AS-48 and facilitate the access to the leader peptidase/cyclase enzyme(s) (see top alignment of Fig. 1a). The diversity of amino acids at the −1 position and the lack of expression of the mutant AS-48 H-1I could indicate a preference in this position for polar or even negatively charged amino acids over hydrophobic residues except in the circularin A homologues.

The identification of new gene clusters encoding for circular bacteriocins shows that often the homologues of As-48D_1_EFGH (immunity determinants) are not found in the gene clusters. The opposite orientation of the *as-48FGH* homologues and the fact that *as-48D_1_EFGH* homologues have their own promoter in addition to the promoter P_2_ upstream *as-48C_1_* [55] suggests that this transporter could have been acquired later during evolution. The knock-out mutants of As-48EFGH were able to produce AS-48, although they showed increased sensitivity [35]. It has been reported that in garvicin ML mutants lacking the homologous genes showed no increased sensitivity and no clear function could be assigned to them in the production of garvicin ML [50]. In the latter case, the presence of an As-48D_1_ homologue and the immunity conferred by the PF01944 containing protein [36] resulted in wild-type level immunity. Our data show that *B. pumilus* B4107 is sensitive to AS-48 and pumilarin. This could potentially be due to the lack of As-48D_1_EFGH homologues. The sensitivity of the pumilarin-producing strain is similar to that of the other *B. pumilus* strain tested and the other *Bacillus* species in general. This is clearly in contrast to *Enterococcus faecalis* JH2-2 (pAM401-81), which is resistant to high concentrations of purified AS-48 [35]. These data suggest that the expression of additional immunity determinants in *B. pumilus* B4107 could be a feasible strategy to increase the production level of pumilarin in the same way as it has been achieved with subtilin [56].

The primary structure and charge distribution in pumilarin is very similar to that of AS-48; therefore, a common mechanism of action is expected. AS-48 DF-I structure is formed by two protomers with a strong dipolar moment that approximate the membrane of the target cell due to charge interactions. This dimer is then disassembled and transforms into the membrane inserted DF-II form to create toroidal pores [22, 23, 57]. The presence of four Glu residues in AS-48 in a plane dividing the charged domain and the hydrophobic core [18] is a feature conserved in pumilarin. Other AS-48-like head-to-tail cyclized peptides keep at least three out of the four negatively charged amino acids, although the one in position 49 (numbered according to AS-48 sequence) is mutated into Gln in some cases. Single replacements of Glu into Ala showed that, in spite of being highly conserved, the negative charge reduction is tolerated although a 2- to 10-fold increase in the MIC was observed [58]. More important is the effect of the mutation Trp24Ala, which caused a dramatic increase in the MIC of AS-48. Pumilarin has an Ala in position 24. We can observe that in this case the MIC compared to AS-48 is not so different, probably due to the additional changes present in pumilarin, which might compensate for the lack of Trp24.

Pumilarin has a relatively broad antimicrobial spectrum, but particularly strong against bacilli. The MIC data presented in this work suggest that pumilarin is evolved in such a way that the specific activity against other bacilli is potentiated. The activity is particularly high against *B. cereus* and *Bacillus subtilis* in the plate assays performed. However, the activity against lactic acid bacteria, *Staphylococcus aureus* or *Listeria monocytogenes* is relatively reduced. Surprisingly, *B. pumilus* B4107 shows sensitivity to both AS-48 and purified pumilarin, which indicates that the self-immunity machinery is not as efficient as that of AS-48. The fact that *Enterococcus faecalis* S-48 is fully resistant to AS-48, whereas *B. pumilus* B4107 is sensitive to both bacteriocins, seems to indicate that there is not full cross resistance between circular bacteriocins in spite of their high similarity. This fact was already observed testing lactocyclicin Q and leucocyclicin Q against the respective producer strains [59]. Interestingly, pumilarin displays some activity against *E. coli* that is not observed for other head-to-tail antimicrobial peptides, indicating that pumilarin could be a good starting point for peptide engineering to obtain highly relevant activity against Gram-negative pathogens.

The study presented here highlights the strength of genome mining approaches for the discovery of novel natural products with high potential. Using this approach, we have identified and compared a large set of gene clusters potentially encoding novel head-to-tail cyclized bacteriocins. As an example, we have isolated and characterized pumilarin, a novel head-to-tail cyclized bacteriocin with a broad antimicrobial spectrum and promising biotechnological properties in food preservation and medicine.

## Data bibliography

1. van Heel AJ, Montalbán-López M, Oliveau Q, Kuipers OP. FigShare, https://figshare.com/s/d318500f36414b4ccfe5 (2017).

## Supplementary Data

559 Supplementary File 1

## Supplementary Data

26 Supplementary File 2

## Supplementary Data

296 Supplementary File 3

## Supplementary Data

232 Supplementary File 4
