# Supplementary material for: Genome-guided identification of novel head-to-tail cyclized antimicrobial peptides, exemplified by the discovery of pumilarin
Source: Microb Genom. 2017 Sep 25;3(10):e000134. doi: 10.1099/mgen.0.000134 (PMC5695211; doi:10.1099/mgen.0.000134)
Supplement: Supplementary File 1 [file mgen-3-134-s001.pdf]

Supplementary material:

```
S. pneumoniae 801                           MNNSIMASEFGLNKIEKAFLFS--LLVSVVTIVAVG-N=IYWVCGKFGIELAPGWYQDIVDFVSSGGTIVEAFAAVAGVTLPAWVGPVLAAFGLASA
Streptococcus sp. 2_1_36FAA                 ---------------------------------MLIAVG-N=IYWICGKFGIRLAPGWYQDIVDFVSAGGSIVDAFAIIAGITLPAWIAPVLAGFGVVSA
B. fibrisolvens AR10 (Butyrivibriocin AR10)    MSKKQIMS--NCISIALLIALIPN=IYFIADKMGIQLAPAWYQDIVNWVSAGGTLTTGFAIIVGVTVPAWIAEAAAAFGIASA
L. (para)casei                              ----------MTKMEKTVLTIGSFVIAALMVTILFTN=IYFIANKLGIHLAPGWYQDMVNYVSAGGSLAGAFSVVAGVTLPAWIVPIATAFGAVSA
                                                                    :   :  * **::..*:**.***.****:*::**:**::. .*: :.*:*:***:     :.** .**
```

Figure S1. Alignment of peptides identified with BAGEL3 that show similarity to Butyrivibriocin AR10 (1). The end of the (predicted) leader is indicated with an = sign. The butyrivibriocin AR10 variants we identified are highly homologous except for the leader sequences.

```
C. saccharolyticus DSM 8903 ---MLF=VAATLGIPETLATTIVNIILTGSTIVSIILALTSILTAGVSAVILAGGWTAFINLVKSKVAQLGLRGAILW
C. bescii DSM 6725          ---MLY=VAATLGIPETLAVTIVNIILTGSTIVSIIIALTSILTAGVSAIILEGGWTAFINLVKSKVAQLGLRGTILW
G. kaustophilus HTA426      MSLLAL=VAGTLGVSQSIATTVVSIVLTGSTLISIILGITAILSGGVDAILEIG-WSAFVATVKKIVAERGKAAAIAW
Geobacillus sp C56 T3       MSLLAL=VAGTLGVSQAVATTVVSIVLTGSTLISIILGITAILSGGVDAILEIG-WSAFVATVKKIVAERGKAAAIAW
B. cereus H308197           -MGLFH=VASKFKVSQGIAGGVVAAVLNAGTLISAIGAVTVVMSGGIDAILEVG-WTAFVATVKEQAAKRGTAGAVAW
B. cereus IS075             -MGLFH=VASKFKISQGIAGGVVAAVLNAGTLISAIGAVTVVMSGGIDAILEVG-WTAFVATVKEQAAKRGTAGAVAW
B. cereus H308197C          -MDLFH=VASKLSISKGVAAGVVSTVLNAGTLITAIGAVTVALSGGIDAILEVG-WATFVATVKEQAAKRGTAGAVAW
B. coagulans XZL9           -MGLFH=VASKFHVSAGIASGVVTAVLHAGTIASIIGAVTVVMSGGVDAILDMG-WTAFIAEVKHLAKEYGKKRAIAW
C. beijerinckii (Circularin A) ---MFL=VAGALGVQTAAATTIVNVILNAGTLVTVLGIIASIASGGAGTLMTIG-WATFKATVQKLAKQS-MARAIAY
C. perfringens E str JGS1987 ---MFD=LAGMLGVDFYAAKKVIDIVATAGTVASVIGIVAAVAGTGG---IGVG----ILAAAKALAKKYGTKAAATW
                               :   :*. : :    *  :: :  ..*: : : ::     *    : *    :   .:  . :     :  :
```

Figure S2. Alignment of peptides identified with BAGEL3 that show similarity to Circularin-A (2). The end of the (predicted) leader is indicated with an = sign.

```
B. thuringiensis IBL 4222     ------MFE=IMGYFGVDRGWATKIVNAIDAVGWGVAAASAIAAIVTAGGLAVTSAMIDVAIFTVKDFLRRNLKAQAVAW
B. thuringiensis HD 789       ------MFE=IMGYFGVDRGWATKIVNAIDAVGWGVAAASAIAAIVTAGGLAVTSAMIDVAIFTVKDFLRRNLKAQAVAW
B. cereus VD184               ------MFE=VMGYFGVDRGWATKIVNAIDAVGWGVAAAGTIAAIISAGGLAVTSAMIDAAIITVKSYLKRNLKAQAIAW
Bacillus sp. 1NLA3E           ------MLE=AMGFFGVGKTLATQIVNVVDAVGYAAIAVSTIMAILSAGGLAPTAAAIDFAIIYIKKKIANNLKAQAIVW
P. larvae B3650               MKGGILLFQ=IAGYLGISTGMAKKIVDVIDAAGWAFVAVSTIMAILSAGGLAVTSAMVDYAIIYVKDLLKRNLKAQAIVW
S. uberis 0140J (Uberolysin)  ---MDILLE=LAGYTGIASGTAKKVVDAIDKGAAAFVIISIISTVISAGALGAVSASADFIILTVKNYISRNLKAQAVIW
                                 :::    *: *:    *.::*:.:*  . .    . * ::::**.*. .:*  *  *: :*. : .******: *
```

Figure S3. Alignment of peptides identified with BAGEL3 that show similarity to Uberolysin (3). The end of the (predicted) leader is indicated with an = sign.

Supplementary Table 1. Strains used in this work.

Strain	Relevant properties	Reference
B. pumilus B4107	Pumilarin producer	Genome announcement
B. pumilus KMM62	Indicator strain	Laboratory collection
B. subtilis 168	Indicator strain	Laboratory collection
E. faecalis VE14089	Indicator strain	Laboratory collection
E. faecalis JH2-2	Indicator strain	Laboratory collection
E. faecalis V583	Indicator strain	Laboratory collection
S. pneumoniae D39	Indicator strain	Laboratory collection
M. flavus B423	Indicator strain	NIZO food research

L. monocytogenes	Indicator strain	Laboratory collection
B. cereus ATCC14579	Indicator strain	Laboratory collection
S. aureus RN6390B	Indicator strain	Laboratory collection
E. coli DH5a	Indicator strain	Laboratory collection
Lactococcus lactis NZ9000	Indicator strain	Laboratory collection
Mycobacterium smegmatis	Indicator strain	Laboratory collection
S. aureus MRSA CAL	MRSA Indicator strain	Girbe Buist UMCG
S. aureus MRSA MW2	MRSA Indicator strain	Girbe Buist UMCG

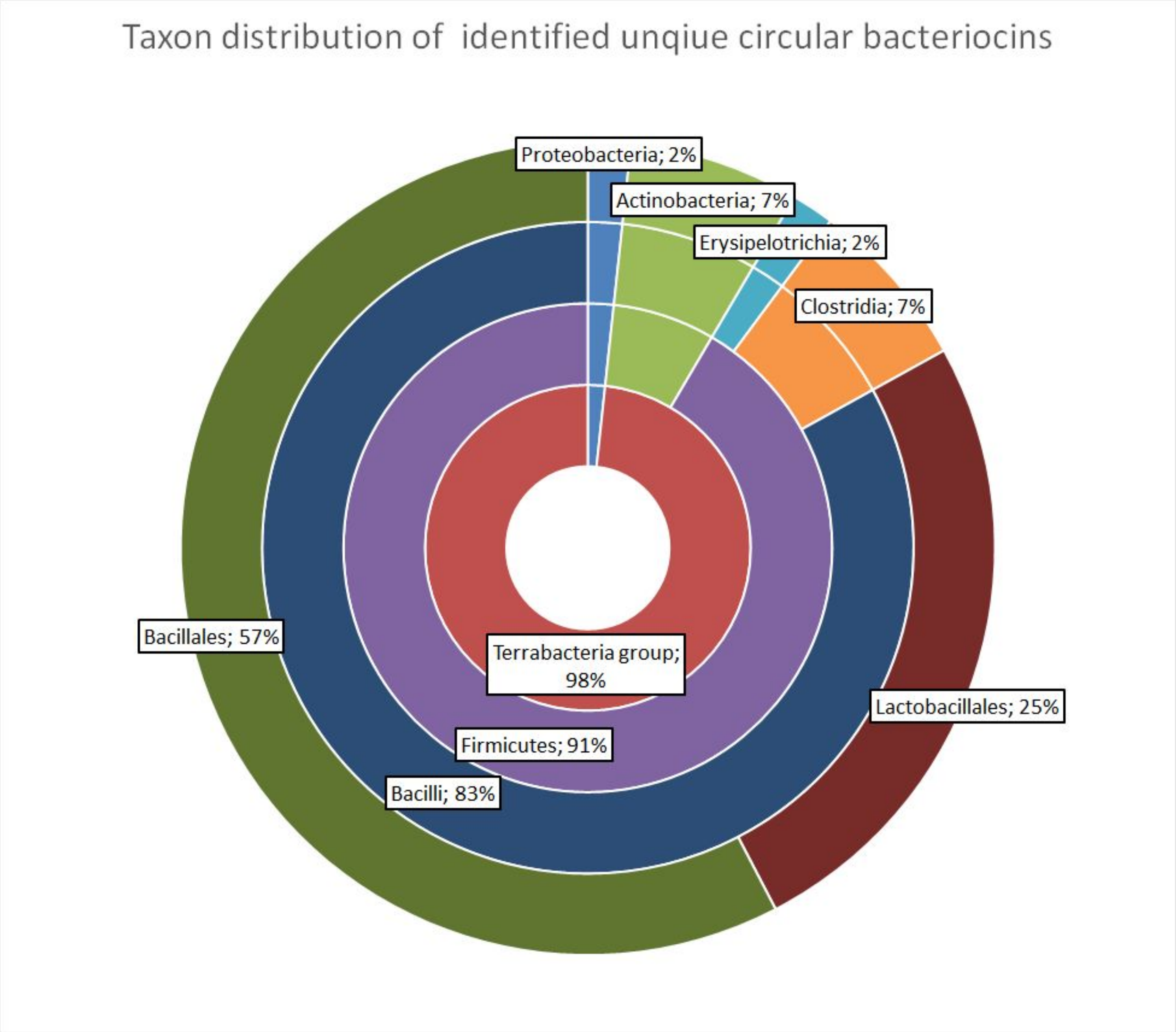

Figure S4. Distribution of the identified circular bacteriocins over the different taxa. In total 59 unique (at least one AA difference in the leader or core peptide) sequences were identified.

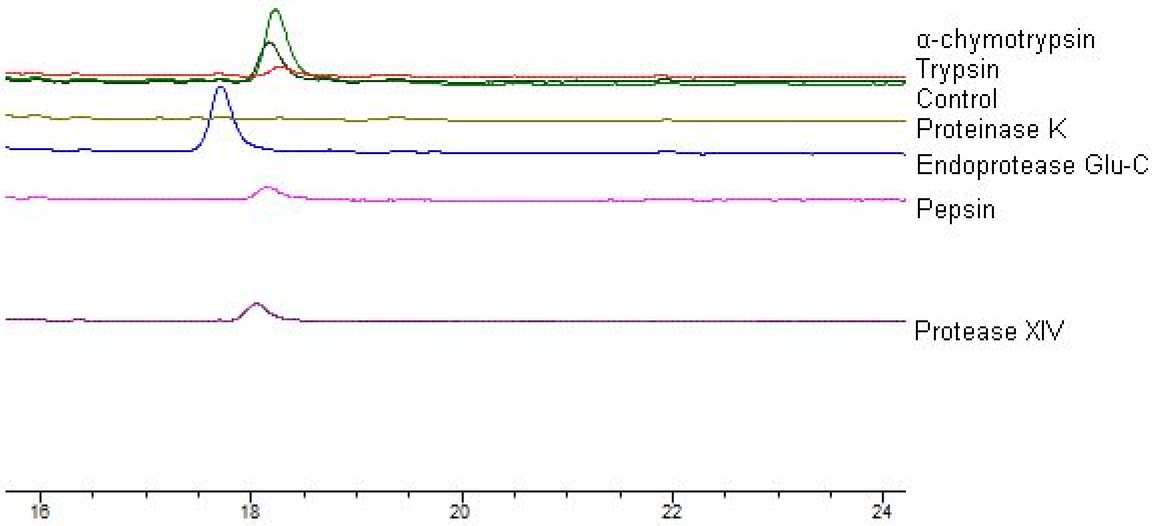

Figure S5. HPLC profile at 280 nm of pumilarin (control) and pumilarin cleaved with different proteases. Light green corresponds to the uncleaved peptide, dark green to the proteolysis with trypsin, red with α-chymotrypsin, brown with proteinase K, blue with endoprotease Glu-C, pink with pepsin and purple with protease XIV. The separation was performed according to (4).

1. Kalmokoff ML, Cyr TD, Hefford MA, Whitford MF, Teather RM. Butyrivibriocin AR10, a new cyclic bacteriocin produced by the ruminal anaerobe *Butyrivibrio fibrisolvens* AR10: characterization of the gene and peptide. Can J Microbiol. 2003 Dec;49(12):763–73.

2. Kemperman R, Kuipers A, Karsens H, Nauta A, Kuipers O, Kok J. Identification and characterization of two novel clostridial bacteriocins, circularin A and closticin 574. Appl Environ Microbiol. 2003 Mar;69(3):1589–97.

3. Wirawan RE, Swanson KM, Kleffmann T, Jack RW, Tagg JR. Uberolysin: a novel cyclic bacteriocin produced by *Streptococcus uberis*. Microbiology. 2007 May;153(Pt 5):1619–30.

4. Montalbán-López M, Spolaore B, Pinato O, Martínez-Bueno M, Valdivia E, Maqueda M, et al. Characterization of linear forms of the circular enterocin AS-48 obtained by limited proteolysis. FEBS Lett. 2008 Sep 22;582(21-22):3237–42.
